# Examination of Submental Space as an Alternative Method of Airway Assessment (Submental Sign)

**DOI:** 10.1186/1756-0500-4-221

**Published:** 2011-06-29

**Authors:** Mihan J Javid

**Affiliations:** 1 Department of Anesthesiology, Tehran University of Medical Sciences, Imam Khomeini medical center, End of Keshavarz Boulevard, Tehran, Iran

## Abstract

**Background:**

Difficult airway especially failed intubation has been associated with a high incidence of mortality and morbidity. Most of mortalities occur when an anaesthesiologist encounters an unanticipated difficult airway.

**Findings:**

In 1999, a 23 yr. old, 65 kg weight and 170 cm height female patient had been scheduled for arthroscopy. Despite totally normal airway assessment (thyromental distance, mouth opening, jaw and neck movement ...) I was astonished by encountering a grade IV Cormack - Lehane laryngoscopic view. Tracheal intubation was impossible and ventilation was very difficult.

On attempt to attain a better laryngoscopic view, while manipulating submandibular region I encountered a bulky noncompliant submental space (Submental Sign). This event made me more alert regarding this finding. Thereafter I noted for this sign throughout the past years and I found it very helpful.

These findings encouraged me to write this report, and suggest a routine examination of submental space in order to keep the safety of the patient at the heart of the care we provide.

**Conclusion:**

Evaluation of the submental space is suggested as an alternative predictor of difficult airway and routine examination of the submental space is of value in airway assessment.

## Discussion

Most anaesthesiologists have experienced difficult airway management. This event is the most critical and stressful situation that may be faced in the practice of anaesthesia.

Serious complications occur especially when mask ventilation is difficult or impossible. If the anaesthesiologist can predict which patients are likely to be difficult for intubation, the risk of anaesthesia reduces considerably [1].

There are various clinical techniques to predict difficult intubation. Airway assessment indicators vary from the simple indicators which often fail to predict difficult airway, to complex, which are not practical. None have been reliable enough to predict airway problems [2].

The physical characteristics associated with difficult intubation include obesity [2-7], limited head and neck [2,5-9]and jaw movement [2-4], receding mandible [2-5,8], long upper incisors [2-4,7], Mallampatti scores [2,4-9], maxillary incisor characteristics [2,5], decreased mouth opening [6-8], shortened thyromental distance [5,6,8,9], short neck [2,5] and limited mandibular protrusion [10,11]

Sometimes despite normal physical characteristics mentioned above, the anaesthesiologist is faced with an unexpected difficult intubation. Upper airway soft tissue abnormalities may be responsible for some of these unexpected, life threatening difficult intubation and ventilation.

Based on my experience of an unexpected difficult intubation in 1999 in a 23 year old, 65 kg weight and 170 cm height female patient scheduled for arthroscopy I decided to evaluate submental space in all of patients.

In the case mentioned above, despite the absence of clinical criteria indicating difficult airway, in direct laryngoscopy I faced with a laryngoscopic view of class IV in the Cormack - Lehane classification. Intubation was impossible and ventilation was very difficult.

While touching and pressing the thyromental area in order to provide a better laryngoscopic view of larynx, I encountered a noncompliant compact bulk instead of a normal soft fatty tissue in the submental space.

After this event I focused on the submental area in all patients before laryngoscopy.

Observation, palpation and evaluation of the submental space for more than ten years, as a routine examination in about 3 thousands patients with no skeleto-facial abnormality resulted in suggesting evaluation of the submental space as a routine examination before laryngoscopy in order to predict unexpected cases of difficult intubation.

This study was conducted in 3000 patients scheduled for general anaesthesia.

The study was started following obtaining written informed consent. A separate written informed consent was obtained for publishing the photographs.

During a ten year study, submental space was routinely examined in all patients.

Patients with other positive standard criteria for difficult intubation were excluded.

## Technique of examination

Examination of the submental sign was performed in all of the patients in a supine position and neutral position of the head. Patients were asked to breathe deeply and they were evaluated during a deep expiration for a bulky noncompliant submental space.

During my routine assessment of airway, I noted that hyoid bone was easily palpable and detectable beyond a compliant submental space. The noncompliant submental space was accompanied by non palpable hyoid bone.

After the evaluation of the submental sign, the grade of laryngoscopic view in the Cormack-Lehane classification was recorded.

Finding a non-compliant submental space (positive submental sign) is indicative of difficult intubation and a compliant submental space (negative submental sign) is indicative of easy intubation.

Difficult intubation is defined as a Cormack-Lehane grade of III and IV and easy intubation is defined as a Cormack-Lehane grade of I and II in laryngoscopic view.

## Findings

Routine examination of the submental space during more than 10 years, in 3000 patients, showed that in the absence of bony structure abnormalities, normal compliance of submental tissue indicated a straight forward intubation, while palpation of a noncompliant bulk in the submental space indicated III or IV laryngoscopic grade in the Cormack - Lehane classification. Palpation of a bulged, noncompliant bulk indicated an impossible intubation. In the latter situation mask ventilation was too difficult.

On further detailed examination I noted that a positive submental sign is associated with a reduced hyomental distance.

During this 10 year study I detected 32 cases of positive submental sign. All of these patients had difficult intubation and compromised airway.

**Figure 1:**
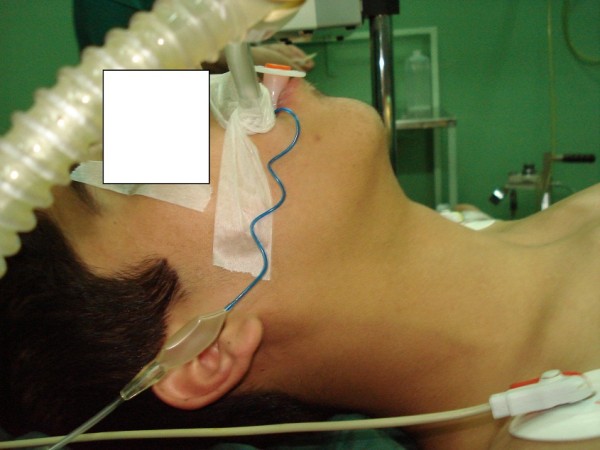
**Normal submental space**.

**Figure 2:**
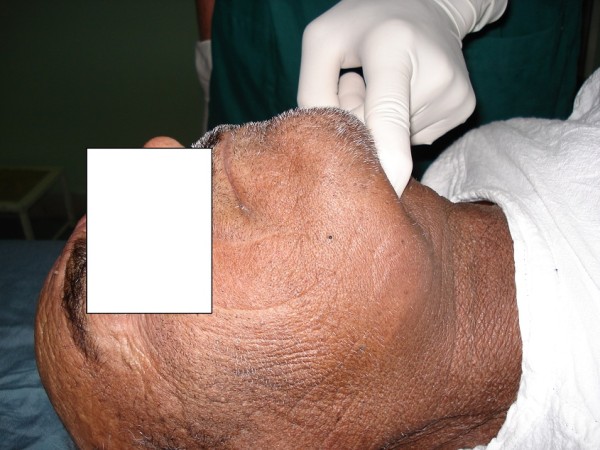
**Compliant submental tissue**. Negative submental sign.

**Figure 3:**
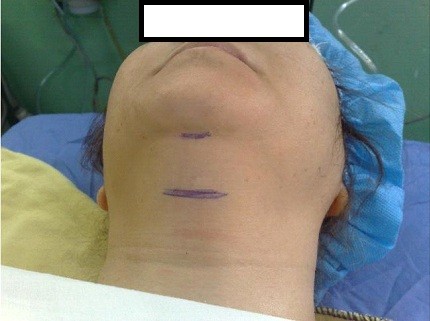
**A bulky submental tissue (type A)**.

"Submental sign" (SMS) is defined as a bulky non-compliant submental space. In a normal patient submental space consists of a thin layer of adipose tissue with a deep curve which is easily compressible in palpation (Figure 1 and 2) and the hyoid bone and laryngeal cartilages are sharply palpable but when the submental sign is positive, submental space consists of a noncompliant, bulky tissue and the hyoid bone and laryngeal cartilages are not easily palpable. (Figure 3)

Submental sign can be classified as:

1- Type A or moderate positive submental sign defined as a bulky noncompliant submental space with direct laryngoscopic view of Cormack- Lehane grade III.

**Figure 4:**
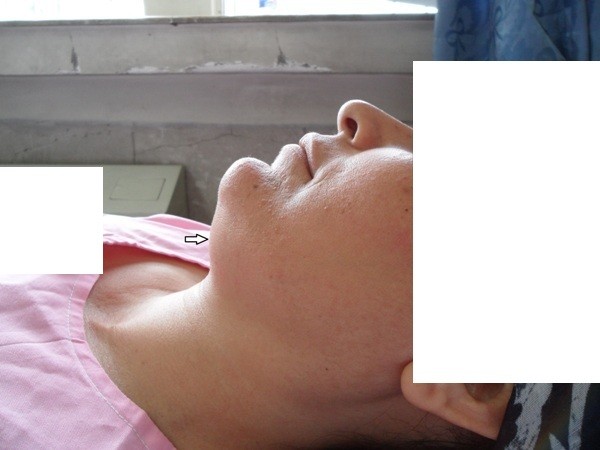
**A bulky bulged submental tissue (type B)**.

2- Type B or severe positive submental sign defined as a bulky bulged noncompliant submental space with a direct laryngoscopy view of Cormack-Lehane grade IV. (Figure 4)

Although Submental sign is a qualitative sign but there is a close relation between the submental sign and hyomental distance. In a normal patient hyomental distance is more than 3 fingers and there is a deep submandibular curve. Positive submental sign is accompanied by a hyomental distance less than 2 fingers and submandibular curve becomes significantly shallow. Submental sign (SMS) indicates a significant anterior placement of larynx.

Examination of thyromental distance has been used to assess submandibular compliance but it has been evaluated as having limited value to predict difficult laryngoscopy [12] on the other hand submandibular compliance has been mentioned for airway assessment. [13]

During this study, in all patients with positive submental sign tracheal intubation was difficult or impossible.

20 cases had moderate submental sign in whom tracheal intubation was very difficult and 12 patients had severe positive submental sign and tracheal intubation was impossible.

## Advantages of submental sign

-Easy prediction of difficult intubation

-Easy to remember

-Suitable to perform on obtunded and or non cooperative patients

-Suitable to use in Emergency ward

## Limitations of this study

Submental sign (SMS) is intrinsically a qualitative predictor but there is a close relation between positive submental sign and reduced hyomental distance.

Although I found submental space assessment of value, it would need to be evaluated in comparison to other predictors of difficult intubation and more investigations are necessary to define specificity and sensitivity of the Submental Sign.

## Conclusion

Submental sign is an easy and practical sign to predict difficult tracheal intubation and the evaluation of submental space by routine examination of the submental space is suggested as a lifesaving examination.

## Consent section

The study was started following obtaining written informed consent. A separate written informed consent was obtained for publishing the photographs.

## List of abbreviations

SMS means sub-mental sign and is defined as a bulky non-compliant submental space.

## Competing interests

The author declares that they have no competing interests.
